# Physicians’ knowledge, attitude, and prescribing behavior regarding stress ulcer prophylaxis in China: a multi-center study

**DOI:** 10.1186/s12876-021-01979-z

**Published:** 2021-10-25

**Authors:** Xiao Xuan Xing, Chen Zhu, Yan Qi Chu, Xiang Rong Bai, Ke Wang, Si Tao Zhang, Su Ying Yan

**Affiliations:** 1 Department of Pharmacy, Xuanwu Hospital of Capital Medical University, No. 45, Changchun Street, Xicheng District, Beijing, People’s Republic of China; 2 National Clinical Research Center for Geriatric Disorders, Beijing, People’s Republic of China; 3 Sir Run Run Shaw Hospital, School of Medicine, Zhejiang University, Hangzhou, People’s Republic of China

**Keywords:** Stress ulcer prophylaxis, Prescribing behavior, Multi-center study, Surgeons

## Abstract

**Background:**

Perioperative patients are at risk of developing stress ulcers (SU), which can cause clinically important bleeding. Stress ulcer prophylaxis (SUP) is widely applied to the patients in Intensive care unit (ICU) as well as the general ward, so it may lead to overmedication. However, there have been no surveys regarding SUP knowledge or prescribing habits.

**Objective:**

Our study assessed the knowledge, attitudes, and prescribing behavior of the surgeons toward perioperative patients regarding SUP and determined factors associated with low knowledge and high level of prescribing behaviors.

**Methods:**

We performed a cross-sectional survey using questionnaires, randomly sampling 1266 surgeons on their current SUP practices.

**Results:**

Proton pump inhibitors for SUP were used the most (94%); 43% used lansoprazole. Guideline awareness was inconsistent; the most familiar guideline was the National Medical Journal of China, and 46% were unaware of any guidelines. The predictors of low knowledge score regarding SUP in multivariable analysis were the hospital grade (p = 0.000), the type of hospital (p = 0.044), attendance at continuing education programs (p = 0.037), the awareness of clinical practice guidelines (CPGs) for SUP (p = 0.000). Twenty-one percent of physicians were high prescribers. High prescribing behavior was associated with hospital grade(p = 0.000), education level(p = 0.010) and attendance at continuing education programs (p = 0.000).

**Conclusion:**

We found that most surgeons used SUP, primarily proton pump inhibitors. However, surgeons knew little about the SUP guidelines, which may lead to insufficient SUP knowledge and overmedication. In addition, hospital grade, the type of hospital and attendance at continuing education programs may also affect the low knowledge of SUP. Hospital grade, education level and attendance at continuing education programs may affect high prescribing behavior.

## Background

Perioperative patients are at risk of developing stress ulcers (SU), which can cause clinically important bleeding (CIB). Treatment is generally by stress ulcer prophylaxis (SUP) via proton pump inhibitors (PPIs) and histamine-2 receptor blockers (H_2_RAs) [1, 2]. However, in recent years, CIB has rarely been reported, yet some publications suggest that SUP was used in three-quarters of critically ill patients worldwide [3, 4]. For example, a recent observational study involving 1034 patients in 97 ICUs across 11 countries only reported clinically important gastrointestinal bleeding in 2.6% of patients [3], but PPI use for SUP has increased in surgery patients [4].

Despite the patients receiving SUP (primarily with PPIs), the efficacy and safety remain controversial. Recent studies comparing SUP versus a placebo treatment or no prophylaxis in critically ill patients highlighted the lack of evidence to support SUP use [5–8]. A large multi-center randomized controlled trial compared pantoprazole and placebo SUP treatments and, unexpectedly, showed that pantoprazole did not reduce mortality or improve the composite secondary outcome [8].

Organizations, such as The American Society of Health-System Pharmacists (ASHP), the Danish Society of Intensive Care Medicine, the Danish Society of Anesthesiology and Intensive Care Medicine (DSAICM), the Eastern Association for the Surgery of Trauma (EAST), and the Chinese Medical Association Surgery Society (CMASS) published SUP guidelines and agreed that SUP should only be administered to patients with one of the following risk factors: coagulopathy (platelets < 50 × 10^9^/L, international normalized ratio > 1.5), mechanical ventilation for > 48 h, Glasgow coma score ≤ 10, burns to > 35% of body surface area,, a history of gastrointestinal bleeding, or cancer [9–11]. In addition, the CMASS recommend major surgery (lasting more than 3 h) as risk factor for SUP.

Koczka et al. assessed awareness of SUP and suggested that a fear of legal repercussions and ignorance regarding the side effects of acid-suppressive therapy were strongly associated with high levels of prescribing SUP. Hussain et al. also suggested that gastric acid suppressant misuse continues to occur, even by the fellows [12, 13]. As far as we know, there are no studies about the awareness of SUP clinical practice guidelines, and the factors of high prescribing behavior have not been explored thoroughly.

In addition, these studies are limited by small sample sizes and focus only on ICU physicians. Our study assessed the knowledge, attitudes, and prescribing behavior of surgeons regarding SUP and determined factors associated with high level of SUP prescribing behaviors.

## Methods

### Survey design and administration

A panel of surgeons, epidemiologists, and clinical pharmacists from Xuanwu Hospital, Beijing, China, designed the survey. We conducted both pilot testing and the validity and reliability assessments of the survey. We invited 30 colleagues with methodologic and clinical expertise to evaluate the content validity of our instrument on a scale from 1 ‘not favourable at all’ to 5 ‘highly favourable’. Results of the clinical sensibility testing using mean scores on the five-point scale suggested that the instrument had content validity (4.8), face validity (4.7). Cronbach's α coefficient and split half reliability were used to measure the reliability of the questionnaire. Cronbach's α coefficient of the whole questionnaire was 0.782, and Cronbach's α coefficient of each dimension was 0.724–0.784. The split-half reliability of the whole questionnaire in this study is 0.773, and the split-half reliability of each dimension is between 0.714 and 0.779. Therefore, this questionnaire has good validity and reliability.

The electronic questionnaires were e-mailed to 29 sub-center heads. The informed consent form and questionnaires were printed and randomly assigned to physicians having indicated their willingness to contribute to the surveys by clinical pharmacists. The study was a survey on the knowledge, empiric therapy and attitude of SUP, all data were collected without concrete reference to physicians or patients and evaluated anonymously. Ethical committee approval was neither required nor recommended by Xuanwu Hospital Ethics Committee at the time the survey was performed. All methods were carried out in accordance with relevant guidelines and regulations.

We used an anonymous, structured choice questionnaire. The questionnaire consisted of 18 questions in two parts. The first part sought basic demographic details for respondents: gender, age, hospital grade(secondary hospitals, tertiary hospitals), education level (Bachelor's degree, master, doctorate), years of experience(≤ 5 years, 6–10 years, 11–15 years, > 15 years), job title (resident, fellows, consultant), attendance at continuing education programs (yes, no), type of hospital (governmental, private), department (general surgery, urology, neurosurgery, cardiac surgery, thoracic surgery, others) and the second part asked for information on:

- Knowledge of SUP guidelines
- frequency of SUP medicine use
- choice of SUP medicines
- attitude toward SUP

To determine physicians' perceptions regarding the knowledge of SUP guidelines, participants were asked to choose which of the following SUP clinical practice guidelines (CPGs) they could master. Furthermore, the participants were provided with a list of SUP risk factors (mechanical ventilation, coagulopathy, major surgery, history of gastrointestinal bleeding, cancer, high-dose corticosteroids). For each, they were asked to indicate if they considered the factor to be a risk factor to SU (yes/ no).

The frequency of SUP medicine use were determined by asking physicians to indicate how often do postoperative patients received SUP (0–20%, 20–40%, 40–60%, 60–80%, 80–100%) and how often do patients discharged from the hospital remain on SUP(0–20%, 20–40%, 40–60%, 60–80%, 80–100%). ‘A superior physician criticized me because I did not use SUP’ was configured as yes/no.

The choice of SUP medicines was chose in response to the question ‘Which medication would you prescribe for stress ulcer prophylaxis?’(omeprazole, pantoprazole, lansoprazole, esomeprazole, famotidine, cimetidine, ranitidine).

The attitude in referring the physicians for SUP was measured on a scale from 1 ‘not favourable at all’ to 5 ‘highly favourable’ in response to the question ‘Acid-suppressing drugs are useful for SUP’, ‘I worry about that patients may develop gastrointestinal bleeding without SUP’’. ‘A fellow’s request for SUP influenced my decision making’, ‘I agree that SUP is a prescribing habit’ and ‘I perceive proton pump inhibitors(PPIs) as harmless, which influences my decision making’ the answers provided for this statement was ‘a. Always b. Usually c. Sometimes d. Rarely e. Never’ as now we agreed that the answer should be yes or no, Always/Usually/Sometimes considered yes and Rarely/Never considered no.

### *Sample size* calculation

The sample size was calculated according to the formula [14]: n ≥ (k/α)2p(1-p), When the confidence is 1–0.05 = 0.95, α = 0.05 and K = 1.96, p = 0.5, the sample size was 380. Our study meets the sample size.

A low knowledge of SU risk factors was categorized into median for evaluation. A high frequency of SUP prescribing behavior for perioperative patients was categorized into quintiles for evaluation [15–17].

### Data analysis

Data were recorded using an electronic spreadsheet (Microsoft Excel, Microsoft). Univariate and multiple logistic regression analyses were performed to test the association between the predictor variables and low knowledge of SU risk factors and high frequency of SUP prescribing behavior. Statistical significance was set at p < 0.05. All statistical analyses were performed using SPSS software (version 23.0, IBM, USA).

The independent variables were sociodemographic factors, including the hospital grade (secondary hospital, tertiary care hospital), sex (male, female), education level (Bachelor's degree, master, doctorate), years of experience(≤ 5 years, 6–10 years, 11–15 years, > 15 years), job title (resident, fellows, consultant),attendance at continuing education programs (yes, no), type of hospital (governmental, private) and the awareness of CPGs for SUP (yes, no). These variables were been included in the logistic regression.

## Results

There were 1266 questionnaires distributed, and all were completed (164 women and 1102 men, 100% response rate). Most participants were from tertiary hospitals (91%, [n = 1149]), had a master’s degree or higher (75%, [n = 947]), and were fellows or resident surgeons (75%, [n = 944]).

Table1 summarizes the drug selection characteristics. PPIs were chosen the most (94%, [n = 1202]), more specifically, lansoprazole (43%, [n = 544). Table 2 summarizes the awareness of SUP clinical practice guidelines (CPGs); the awareness rate was inconsistent (7–42%). The most familiar guideline was the National Medical Journal of China (NMJC, 42%), but 46% of surgeons were unaware of any guidelines.

**Table 1:** Reported use of SUP medications in practice

	No (%) of respondents
*Agents for SUP*	
Omeprazole	193 (15%)
Pantoprazole	137 (11%)
Lansoprazole	549 (43%)
Esomeprazole	323 (26%)
Famotidine	23 (2%)
Cimetidine	21 (2%)
Ranitidine	20 (2%)

**Table 2:** SUP clinical practice guideline awareness

Guideline	Institutions	No. (%) of respondents*
Consensus review for stress ulcer prophylaxis and treatment	CMASS	530 (42%)
Therapeutic guidelines on stress ulcer prophylaxis	ASHP	138 (11%)
Practice management guidelines for stress ulcer prophylaxis (2008)	EAST	109 (9%)
Guideline for stress ulcer prophylaxis in the intensive care unit	DSAICM and DSICM	85 (7%)
No awareness	N/A	579 (46%)

CMASS, Chinese Medical Association Surgery Society; ASHP, American Society of Health-System Pharmacists; EAST, Eastern Association for the Surgery of Trauma; DSAICM, Danish Society of Anesthesiology and Intensive Care Medicine; DSICM, Danish Society of Intensive Care Medicine; N/A, not applicable

^*^Some participants choose more than one option

### Factors affecting the knowledge regarding stress ulcer prophylaxis overuse in China

**Fig. 1:**
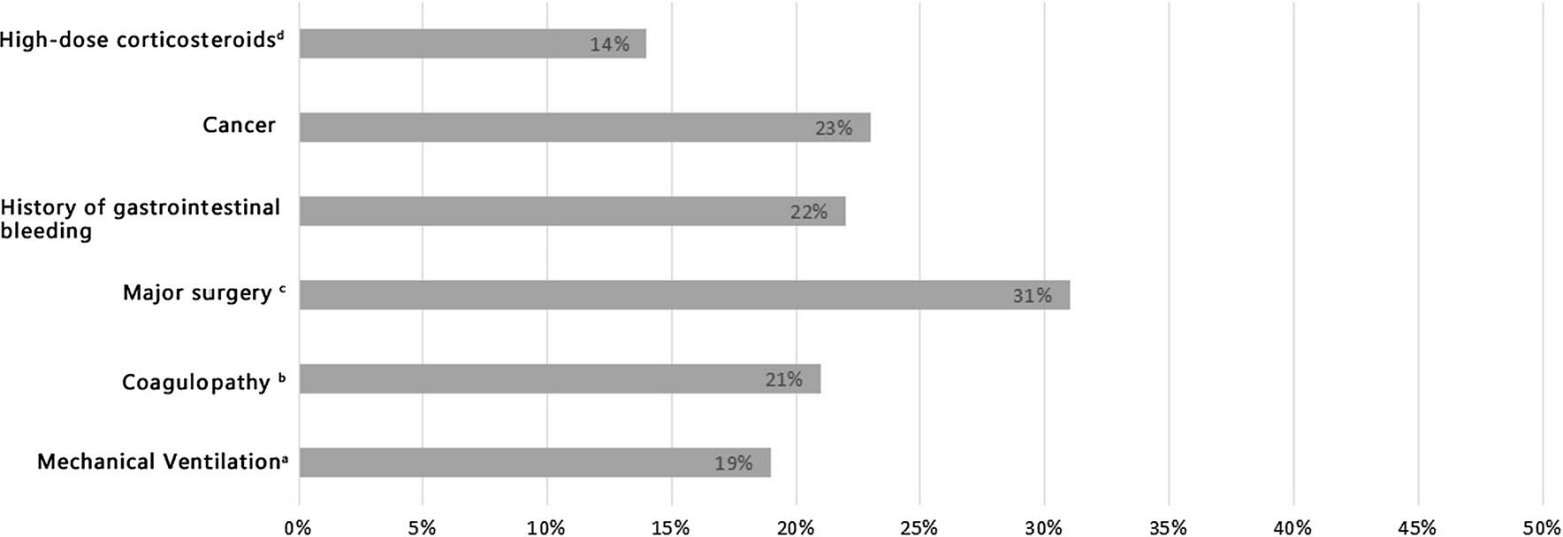
Knowledge about SUP risk factors. ^a^Mechanical Ventilation > 48 h, ^b^Coagulopathy: Platelets < 50 × 109/L, international normalized ratio > 1.5, or prothrombin time > 20 s., ^c^Major surgery > 3 h; ^d^high-dose corticosteroids:50 mg/d of steroids or equivalent methylprednisolone

Figure 1 summarizes the questions and answers regarding SU risk factors. Thirty-one percent of participants (n = 387) knew that major surgery was a risk factor for SUs. Nearly 20% of participants knew that mechanical ventilation, coagulopathy, a history of gastrointestinal bleeding, and cancer were SUP risk factors. However, only 14% knew that administering high-dose corticosteroids increased risk.

For the purposes of the analysis, the knowledge score regarding stress ulcer prophylaxis was categorized as low (< 2) or high (> 2). The predictors of low *knowle*dge score regarding SUP in multivariable analysis were the hospital grade, (odds ratio (OR) 4.187, 95% confidence interval [CI] (2.543–6.894), p = 0.000), type of hospital (OR 0.176, 95% CI 0.033–0.956, p = 0.044), attendance at continuing education programs (OR 0.742, 95% CI 0.561–0.982, p = 0.037), the awareness of CPGs for SUP (OR 0.508, 95% CI 0.394–0.655, p = 0.000) (Table 3).

**Table 3:** Univariate and multivariate analysis of risk factor for low knowledge regarding stress ulcer prophylaxis

Variable	Physicians without low knowledge of SUP	Physicians with low knowledge of SUP	Univariate analysis OR (95% CI)	p value	Multivariate analysis OR (95% CI)	p value
*Hospital grade*						
Tertiary hospitals	370 (87%)	810	3.884 (2.459, 6.134)	0.000	4.187 (2.543, 6.894)	0.000
Secondary hospitals	55 (13%)	31	1.000		1.000	
*Type of hospital*						
Governmental	422 (99%)	835 (99%)	0.658 (0.132, 3.274)	0.609	0.176 (0.033, 0.956)	0.044
Private	3 (1%)	6 (1%)	1.000		1.000	
*Get any continuous educational programs*						
Yes	125 (29%)	177 (21%)	0.640 (0.490, 0.835)	0.001	0.742 (0.561, 0.982)	0.037
No	300 (71%)	664 (79%)	1.000		1.000	
*The awareness of CPGs for SUP*						
Yes	285 (67%)	402 (48%)	0.450 (0.353, 0.574)	0.000	0.508 (0.394, 0.655)	0.000
No	140 (33%)	439 (52%)	1.000		1.000	

Only the statistically significant variables were included in the table

### The attitude regarding stress ulcer prophylaxis

The physicians’ attitudes regarding SUP are listed in Table 4. Most of the participants agreed that acid-suppressing drugs are useful for SUP and that PPIs are harmless. In addition, half of the participants worried that patients may develop gastrointestinal bleeding without SUP. One third of the participants affirmed that an fellow’s request for SUP could influenced decision making. Furthermore, physicians rarely agreed that SUP is a prescribing habit (Table 4).

**Table 4:** Attitudes toward stress ulcer prophylaxis (SUP)

Opinions	5^a^	4	3	2	1
Acid-suppressing drugs are useful for SUP	910 (72%)	238 (19%)	64 (5%)	13 (1%)	41 (3%)
I worry that patients may develop gastrointestinal bleeding without SUP	270 (21%)	362 (29%)	390 (31%)	128 (10%)	116 (9%)

	Yes			No	
A fellow’s request for SUP influenced my decision making	470 (37%)			796 (63%)	
I agree that SUP is a prescribing habit	293 (24%)			973 (76%)	
I perceive PPIs as harmless, which influences my decision making	964 (76%)			302 (24%)	

^a^A five-point Likert-scale ranging from strongly disagree 1 to strongly agree 5 was used to measure the attitude

### Factors affecting the prescribing behavior regarding stress ulcer prophylaxis overuse in China

Eighty-eight percent of participants stated that medications for SUP are used in a vast majority of postoperative patients, and > 80% were classified as having high prescription behavior. Compared with the cases of postoperative patients, only a few participants indicated they would will continue to prescribe SUP for discharged patients who had SUP during hospitalization. These differences are shown in Table 5.

**Table 5:** Stress ulcer prophylaxis (SUP) for postoperative and discharged patients

% of institutions stating that:	% of patients				
	0–20	20–40	40–60	60–80	80–100
Postoperative patients who received SUP	147	302	293	254	268
Hospital discharged patients who remained on SUP	674	295	162	81	54

For purposes of the analysis, SUP prescription was categorized as high (> 80%) or low (< 80%). Twenty-one percent of physicians were high prescribers. High prescribing behavior was associated with hospital grade(p = 0.000), education level (p = 0.010) and attendance at continuing educational programs (p = 0.000). Compared those with a bachelor's degree, the high prescription behavior of individuals with a master's and doctoral degree was 1.685 times and 2.651 times, respectively. The high prescribing behavior of the participants in tertiary hospitals was lower than that in secondary hospitals, and the difference was statistically significant. Similarly, The high prescribing behavior of the participants who attended continuing educational programs was lower than the others (Table 6).

**Table 6:** Univariate and multivariate analysis of risk factor for high SUP prescribing behaviors

Variable	Physicians without high SUP prescribing	Physicians with high SUP prescribing	Univariate analysis OR (95% CI)	p value	Multivariate analysis OR (95% CI)	p value
*Hospital grade*						
Tertiary hospitals	942 (94%)	238 (89%)	0.472 (0.296,0.751)	0.002	0.314 (0.183,0.537)	0.000
Secondary hospitals	56 (6%)	30 (11%)	1.000		1.000	
*Education level*				0.010		0.010
Doctorate	236 (24%)	83 (31%)	1.826 (1.239, 2.692)	0.002	2.551 (1.646, 3.953)	0.000
Master	492 (49%)	133 (50%)	1.404 (0.986, 1.998)	0.06	1.685 (1.136, 2.502)	0.01
Bachelor's degree	270 (27%)	52 (19%)	1.000		1.000	
*Get any continuous educational programs*						
Yes	294 (29%)	8 (3%)	0.074 (0.036, 0.151)	0.000	0.071 (0.034, 0.145)	0.000
No	704 (71%)	260 (97%)	1.000		1.000	

Only the statistically significant variables were included in the table

## Discussion

To our knowledge, this is the first study to evaluate the factors affecting the knowledge, prescribing behavior and the attitudes regarding stress ulcer prophylaxis overuse for perioperative patients in China. Koczka et al. and Hussain et al. [12, 13]. previously described the awareness and attitudes of physicians toward SUP, but most of the literature has only reported the prescribing behavior. Additionally, the factors influencing low knowledge and high prescribing behavior have not been explored, and most participants were ICU physicians, not surgeons.

In this Chinese national survey, we found that 46% of physicians were unaware of SUP guidelines. Additionally, only 7–11% of physicians knew the guidelines for high levels of evidence. Ye [18] assessed CPG quality via Appraisal of Guidelines for Research & Evaluation II, and the overall CPG quality for SUP was relatively low. The DSAICM, ASHP, and EAST [9–11] CPGs were recommended, but the NMJC [19] was not. At present, the university courses related to stress ulcer in China mainly focus on the pathogenesis, clinical manifestations and the treatment of stress ulcer, but do not involve SUP. In our study only 19.7% surgeons learn about continuing educational programs. It is this weakness in the educational process in Chinese universities and problem in the continuing educational programs. Further, the surgeons are busy with surgery, and do not pay attention to SUP.

In prior surveys in China, the SUP agent of choice was PPIs, ranging from 84 to 96% [20, 21]. In our survey, 94% of physicians prescribed PPIs for SUP, supporting recent studies that suggest the growing use of PPIs for SUP [22]. In 2014, Barletta et al. [23] conducted a point prevalence study involving 58 ICUs in the United States and Canada and found that PPIs were the most commonly used agents (70%).

In our survey, some respondents reported that SUP occurred primarily until the patients were discharged from the ICU, but prior observational studies showed that almost 20% of survey respondents indicated that they did not discontinue SUP until the patient was discharged from the hospital [24]. A recent Australian study revealed that 63% of patients continued receiving SUP on the ward without indication, and 39% of patients continued until discharge [25]. These findings are congruent with those of a survey by Krag et al., in which 22% of respondents discontinued SUP upon discharge from the ICU [22].

Our survey results indicated that 21% (n = 268) of respondents often prescribed SUP in postoperative patients. Although we cannot fully ascertain the reasons for a doctor's prescribing behavior through a questionnaire survey, to some extent, we can understand the factors affecting prescribing behavior.

While this study shows that superior hospital grade and attendance at continuing education programs may reduce high prescribing behavior, it is undeniable that doctors in tertiary hospitals have more opportunities to attend continuing education programs than in secondary hospitals. Government agencies should strengthen the training of secondary hospitals, especially the SUP continuing education program. Interestingly, our study found that a higher education level may lead to high prescribing behaviors. However, high prescribing behavior does not mean that prescribing is unreasonable. It may be that these doctors have more critically ill patients after surgery and therefore need SUP.

Recent studies reported low CIB frequency, with the majority of prospective studies and meta-analyses finding little bleeding reduction with pharmacological prophylaxis [8, 26, 27]. For most clinicians, it appears that the value of prophylaxis is overstated, and the presumed benefits of prophylaxis outweigh its associated risks and costs.

There are some limitations in our study. The research area distribution was not balanced, and these views and practices may differ from other physicians. Additionally, our study reflected perceived prescribing practices but did not evaluate actual prescriptions. Regardless, this study had a 100% response rate, which was higher than that of many previous self-report questionnaires [28–31], and is the largest survey of SUP practices and prescribing habits in China.

We found that most surgeons used SUP, primarily proton pump inhibitors. However, surgeons knew little about the SUP guidelines, which may lead to insufficient SUP knowledge and overmedication. In addition, hospital grade, the type of hospital and attendance at continuing education programs may also affect the low knowledge of SUP. Hospital grade, education level and attendance at continuing education programs may affect high prescribing behavior.

## Data Availability

The datasets generated and/or analyzed during the study are available from the corresponding author upon reasonable request.

## Abbreviations

ICU: Intensive care unit
SU: Stress ulcers
SUP: Stress ulcer prophylaxis
CPGs: Clinical practice guidelines
CIB: Clinically important bleeding
PPIs: Proton pump inhibitors
H2RAs: Histamine-2 receptor blockers
ASHP: American Society of Health-System Pharmacists
DSAICM: Danish Society of Intensive Care Medicine, The Danish Society of Anesthesiology and Intensive Care Medicine
EAST: The Eastern Association for the Surgery of Trauma
